# Adiponectin Inhibits Neutrophil Phagocytosis of *Escherichia coli* by Inhibition of PKB and ERK 1/2 MAPK Signalling and Mac-1 Activation

**DOI:** 10.1371/journal.pone.0069108

**Published:** 2013-07-25

**Authors:** Alessandra Rossi, Janet Lord

**Affiliations:** Medical Research Council Centre for Immune Regulation, School of Immunity and Infection, University of Birmingham, Birmingham, United Kingdom; Fundação Oswaldo Cruz, Brazil

## Abstract

Full length adiponectin is a potent immune modulatory adipokine, impacting upon the actions of several immune cells. Neutrophil oxidative burst has been shown to decrease in response to adiponectin, and we speculated that it could have other effects on neutrophil function. Here we report that adiponectin reduces the phagocytic ability of human neutrophils, decreasing significantly the ingestion of opsonised *E. coli* by these cells in whole blood (p<0.05) and as isolated neutrophils (p<0.05). We then determined the mechanisms involved. We observed that the activation of Mac-1, the receptor engaged in complement-mediated phagocytosis, was decreased by adiponectin in response to *E. coli* stimulation. Moreover, treatment of neutrophils with adiponectin prior to incubation with *E. coli* significantly inhibited signalling through the PI3K/PKB and ERK 1/2 pathways, with a parallel reduction of F-actin content. Studies with pharmacological inhibitors showed that inhibition of PI3K/PKB, but not ERK 1/2 signalling was able to prevent the activation of Mac-1. In conclusion, we propose that adiponectin negatively affects neutrophil phagocytosis, reducing the uptake of *E. coli* and inhibiting Mac-1 activation, the latter by blockade of the PI3K/PKB signal pathway.

## Introduction

Adipose tissue is the main source of adipokines, circulating molecules that like cytokines are engaged in regulating a variety of physiological and pathological processes. Adiponectin is the most abundant adipokine, reaching concentrations greater than 10 µg/ml in the circulation [1]. Structurally, adiponectin belongs to the C1q/Tumor Necrosis Factor (TNF) superfamily, with its C-terminal domain sharing homology with the complement factor C1q [2]. Different isoforms of adiponectin have been identified: full-length adiponectin, which further oligomerises to form trimers of low molecular weight, hexamers and polymers of high molecular weight [3].

Adiponectin has aroused increasing interest because of its insulin-sensitising [4], [5], anti-atherosclerotic [6] and anti-inflammatory properties [7] and its levels have been shown to be inversely correlated with obesity [8] and type 2 diabetes mellitus [9], [10]. Adiponectin appears to achieve many of its actions through activation of AMP-activated protein kinase (AMPK), with phosphorylation of AMPK shown to increase following treatment with adiponectin in several cell types including endothelial cells, peripheral blood mononuclear cells (PBMCs) [11] and phagocytes [12]. In relation to its anti-inflammatory role, adiponectin prevents lipopolysaccharide (LPS)-induced acute lung injury (ALI) in mice by inhibiting the production of IL-6 by lung endothelial cells [13] and protects against LPS-induced liver injury in obese mouse models by diminishing TNF-α production [14]. In addition, it has also been shown to inhibit NK cell cytotoxicity [15] and to induce human monocytes to differentiate into alternative the anti-inflammatory M2 macrophage phenotype [16]. Contradictory results have been reported in relation to adiponectin effects on macrophage phagocytosis [17], [18] and dendritic cell function [19], [20].

Neutrophils are the most abundant immune cell population in the blood, representing the first line of defence against microbial pathogens and with a major pro-inflammatory role. These short-lived cells migrate towards the site of infection where they contribute to the removal and the killing of pathogens through the processes of phagocytosis, degranulation and release of microbicidal peptides, production of reactive oxygen species (ROS) and generation of neutrophil extracellular traps (NETs) [21], [22]. Both neutrophil and monocyte ROS production in response to the bacterial product fMLP are reduced by the addition of full-length adiponectin, which inhibits NADPH oxidase activation by decreasing the phosphorylation of the p47phox subunit [12]. In contrast, globular adiponectin has been shown to enhance phagocyte ROS production, favouring NADPH oxidase activation via phosphorylation of the MAPK: ERK 1/2 and p38 [12].

Neutrophil phagocytosis is initiated by ligation of several receptors, including cytokine receptors, pattern recognition receptors (PRRs) such as Toll-like receptor 4 (TLR4), the opsonic Fc-γ receptors FcγRI, FcγRII and FcγRIII (CD16), and the complement receptors CR1 (CD35) and CR3 (CD11b/CD18), alternatively called Mac-1 [23]. Mac-1 undergoes activation by conformational change in stimulated neutrophils thus achieving a higher affinity and avidity towards its ligands [24]. Following binding to neutrophil membranes, bacterial ingestion is associated with intracellular signalling involving MAPK activation: both ERK 1/2 and p38 MAPK are phosphorylated in response to microbial challenge [25], and activation of the PI3K/PKB pathway has also been shown to be fundamental for cytoskeletal rearrangements during phagocytosis [26], [27].

Despite the major pro-inflammatory role of neutrophils the effect exerted by adiponectin on neutrophil phagocytosis has not been investigated, therefore this study aimed to evaluate whether this adipokine could influence the phagocytosis of the bacteria *E. coli* and the mechanisms involved.

## Results

### Adiponectin inhibits neutrophil phagocytosis of E. coli

**Figure 1:**
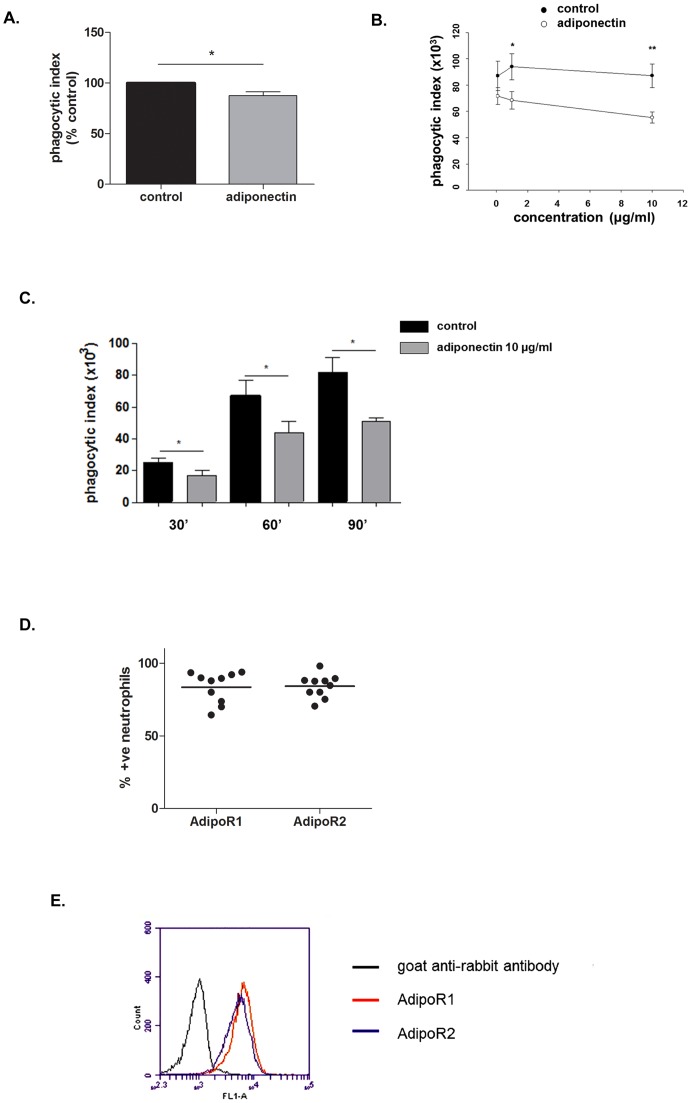
Adiponectin decreases neutrophil phagocytosis. Adiponectin was added to (A) whole blood (n = 10) at a final concentration of 10 μg/ml or (B) isolated human neutrophils (n≥6) at different concentrations (0.1, 1 and 10 μg/ml) prior to addition of opsonized FITC labelled *E. coli* and assessment of phagocytic index by flow cytometry. C. Time course of neutrophil phagocytosis (30, 60, 90 minutes) with and without the presence of adiponectin (10 μg/ml). Data are mean ± SEM and * indicates p<0.05, ** indicates p<0.02. D. Percentage of human neutrophils expressing adiponectin receptors AdipoR1 and AdipoR2. The bar represents the mean value. E. Representative FACS plots are shown for immunofluorescence staining for the adiponectin receptors AdipoR1 and AdipoR2.

Pre-treating whole blood for one hour with a physiological dose of adiponectin (10 μg/ml) resulted in a significant decrease in the neutrophil phagocytic index for uptake of *E. coli* (p<0.05; Fig. 1A). We then confirmed that adiponectin directly modulates neutrophil phagocytosis, using isolated neutrophils in serum-free media pre-incubated with adiponectin for one hour before the addition of *E. coli* (40∶1 ratio, *E. coli*:neutrophils), previously opsonised with autologous serum. Figure 1B shows that adiponectin (0.1, 1 and 10 μg/ml) inhibited neutrophil phagocytosis in a dose-dependent manner. The degree of inhibition was greater than that seen with whole blood possibly due to the presence of adiponectin in the blood. We also determined the stability of the adiponectin effect over time and found the inhibition of phagocytosis was maintained for up to 90 minutes (Figure 1C). Different bacteria to neutrophil ratios (5∶1, 20∶1) were tested and the decrease in phagocytosis with adiponectin was seen at all ratios (data not shown). We also assessed the surface expression of the two receptors for adiponectin, AdipoR1 and AdipoR2, in 10 donors and we consistently found high expression of both receptors (Figure 1D and E).

### Adiponectin reduces Mac-1 activation and bacterial binding to neutrophils

**Figure 2:**
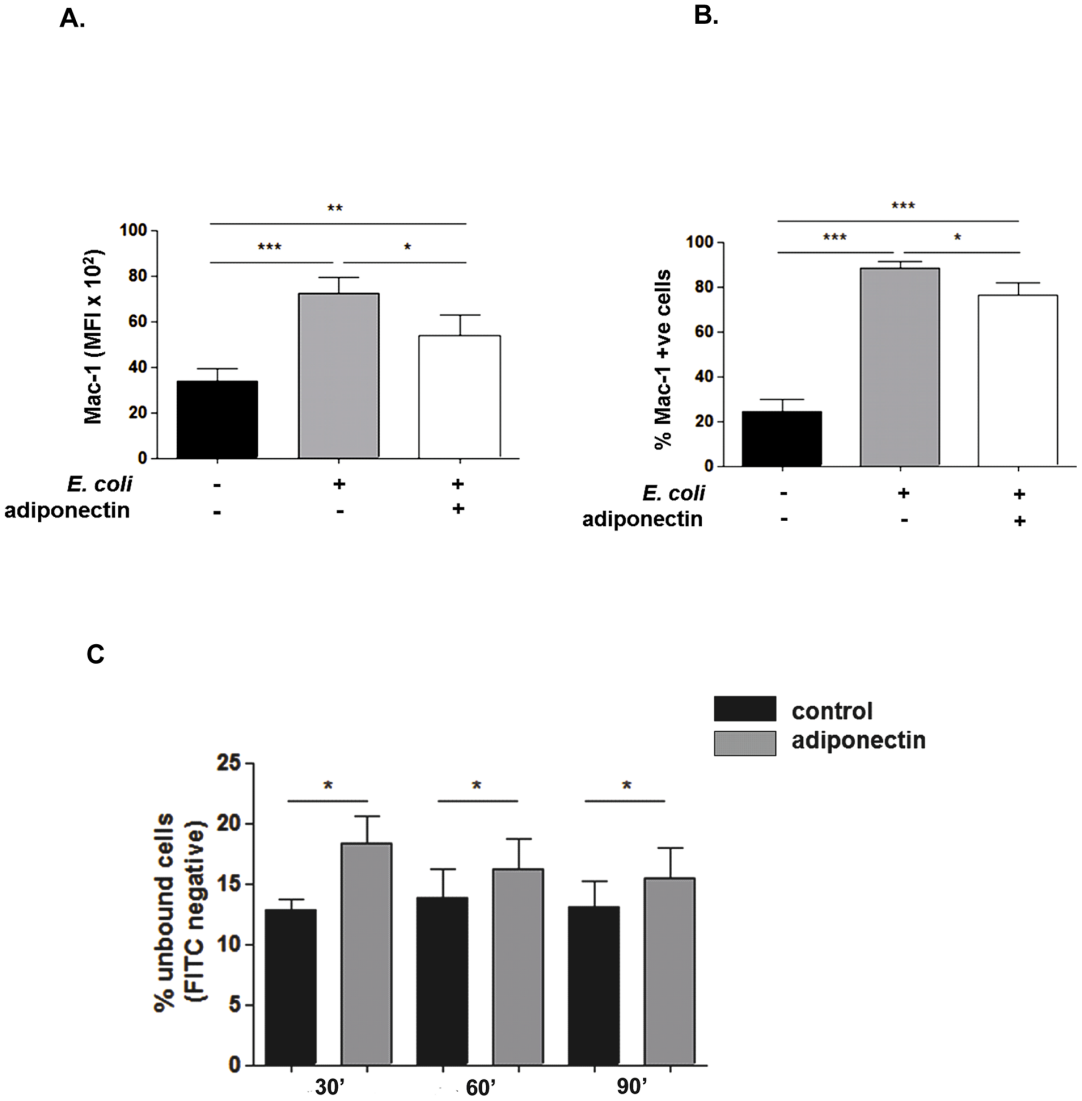
Adiponectin inhibits the activation of Mac-1 and the binding of *E.coli* to the neutrophil surface. Isolated neutrophils treated with adiponectin for 1 hour and stimulated with opsonied *E. coli* were immunostained for the activated conformation of Mac-1. (A) Mac-1 expression (MFI) and (B) the percentage of neutrophils expressing active Mac-1 were decreased by adiponectin. Data are mean ± SEM (n = 7). C. The binding of bacteria to the neutrophil cell surface was measured by flow cytometry following pre-treatment with adiponectin and after 30, 60 and 90 minutes of incubation with opsonized FITC labeled *E. coli* at 4°C. FITC negative cells were considered unbound. Data are expressed as the percentage of cells with no *E.coli* bound and are mean ± SEM (n≥7).

Neutrophils express a broad range of phagocytic receptors which are necessary to promote the ingestion of opsonised microbes. To delineate whether adiponectin could influence the uptake of bacteria through the modulation of phagocytic receptors the surface expression of CD16, CD11b and TLR4 on resting neutrophils was assessed after a one hour treatment with adiponectin. The expression of none of these receptors was affected by adiponectin treatment (data not shown). Mac-1 is a dimer of CD11b and CD18 and is a complement receptor that undergoes activation by conformational change in stimulated neutrophils [24]. Its activation was measured using a specific antibody against the activation induced epitope of Mac-1 after neutrophils were pre-incubated with adiponectin and stimulated with opsonised *E. coli* for 90 minutes. Levels of activated Mac-1 (expressed as MFI, Figure 2A) and the percentage of cells bearing active Mac-1 (Figure 2B) were both significantly decreased in the presence of adiponectin. As the conformational change of Mac-1 leads to increased receptor affinity towards its ligands [24], we hypothesized that the reduction in the phagocytosis mediated by adiponectin may be due in part to decreased binding of bacteria to Mac-1. Therefore, we evaluated the binding of FITC labelled *E. coli* to the neutrophil surface at 4°C at 30, 60 and 90 minutes after pre-incubation with adiponectin. The binding of bacteria was significantly reduced by adiponectin at each time point (Figure 2C).

### Adiponectin inhibits PKB and ERK signalling

**Figure 3:**
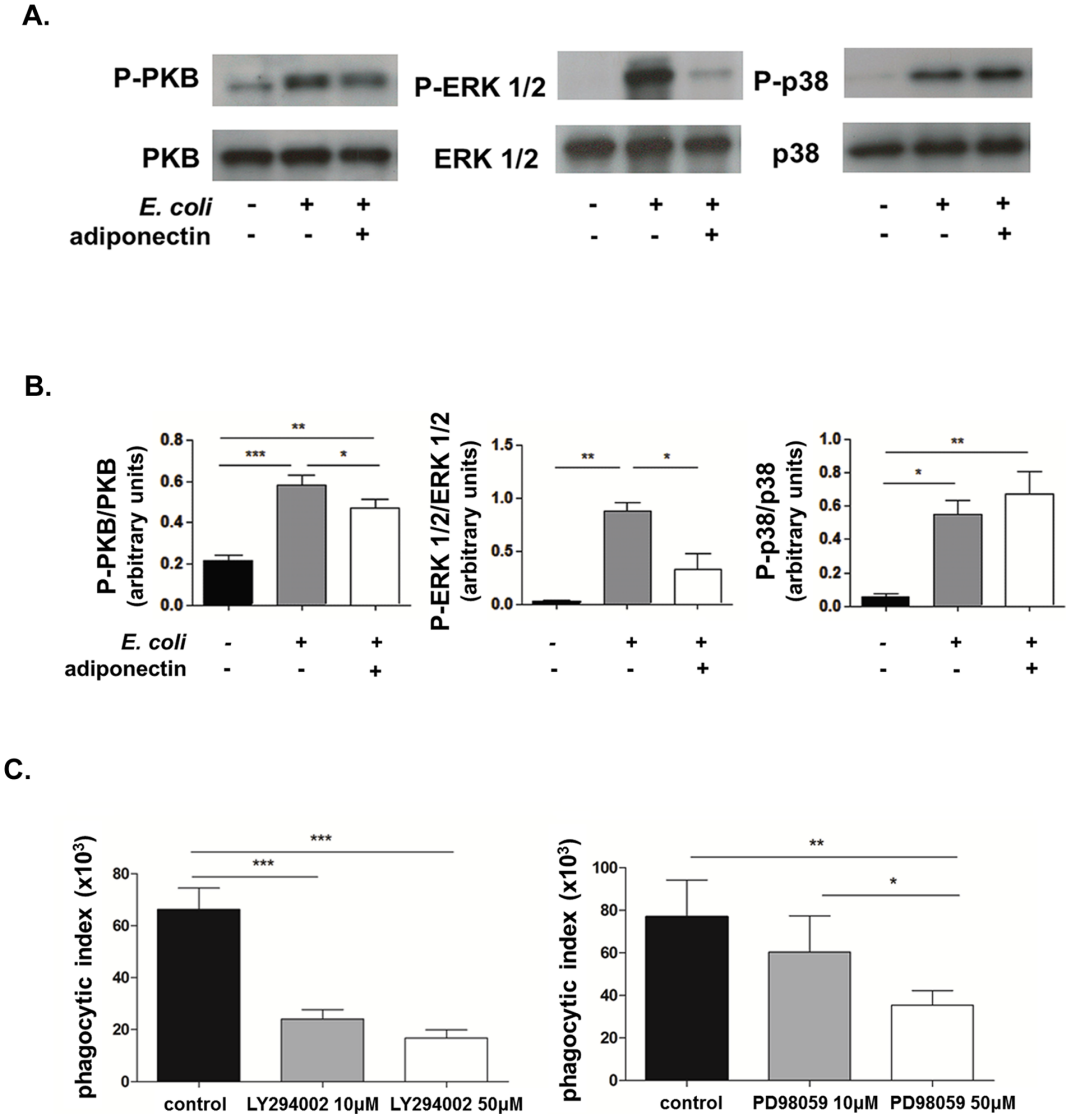
Adiponectin reduces neutrophil phagocytosis by decreasing the phosphorylation of PKB and ERK 1/2 but not p38 in response to *E. coli*. Neutrophils were pre-incubated with adiponectin and stimulated with opsonised *E. coli* for 10 minutes after which proteins were extracted and analysed by western blotting. A. Representative western blots showing the effect of adiponectin on phosphorylation of PKB, ERK 1/2 and p38 MAPK induced by *E. coli*. B. Densitometric analysis of three separate experiments for the effect of adiponectin on phosphorylation of PKB, ERK 1/2 and p38 MAPK, expressed as the ratio between the phosphorylated and unphosphorylated proteins. C. The PI3K inhibitor LY294002 and MEK1 inhibitor PD98059 (10 μM and 50 μM) were added to neutrophils for 30 minutes prior to addition of opsonised FITC labeled *E. coli* and the phagocytic index was measured by flow cytometry. Control samples were treated with the highest concentration of carrier (DMSO) used. Data are mean ± SEM (n = 5). * indicates p<0.05, ** indicates p<0.01, *** indicates p<0.001 for treated versus control cells.

The process of phagocytosis is accompanied by increased activation of PI3K/PKB and MAPK signalling pathways. Hence we evaluated the effect of adiponectin on phosphorylation of PKB, ERK 1/2 and p38 MAPK by western blotting using antibodies specific to the phosphorylated forms of these kinases. *E. coli* stimulated neutrophils which were pre-treated with adiponectin displayed significantly lower phosphorylation of both PKB and ERK 1/2 compared to control stimulated neutrophils, though the effect was consistently most marked for ERK 1/2 (Fig. 3A, B). p38 phosphorylation was not affected by adiponectin.

To confirm that PI3K/PKB and ERK 1/2 activation was necessary to sustain neutrophil phagocytosis, we studied this function after pharmacologically blocking these two pathways. The inhibitors LY294002 (PI3K inhibitor) and PD98059 (an inhibitor of MEKI which is directly upstream of ERK 1/2) were added 30 minutes before initiation of the phagocytosis assay. The inhibition of both PI3K/PKB and ERK1/2 pathways resulted in a reduction in neutrophil phagocytosis (Fig. 3C) in a concentration dependent manner and the efficacy of the two inhibitors was confirmed by western blot (data not shown). The specificity of LY294002 and PD98059 for PI3K and ERK 1/2 is very good and has been reported previously with LY294002 only inhibiting additionally casein kinase 2 even when used at 50μM [28], [29]. The concentrations employed here are also widely used [30]–[34].

As adiponectin signalling results in the activation of AMPK in a variety of cell types, including phagocytes [12], we also evaluated whether this kinase could contribute to decreased neutrophil phagocytosis. We pre-incubated neutrophils with the AMPK activator AICAR (1 mM) for 30 minutes prior to the addition of *E. coli* but the treatment did not reduce neutrophil phagocytosis (data not shown) therefore adiponectin is unlikely to act through this factor to inhibit neutrophil phagocytosis.

### Mac-1 activation depends on PI3K but not ERK 1/2 activation

**Figure 4:**
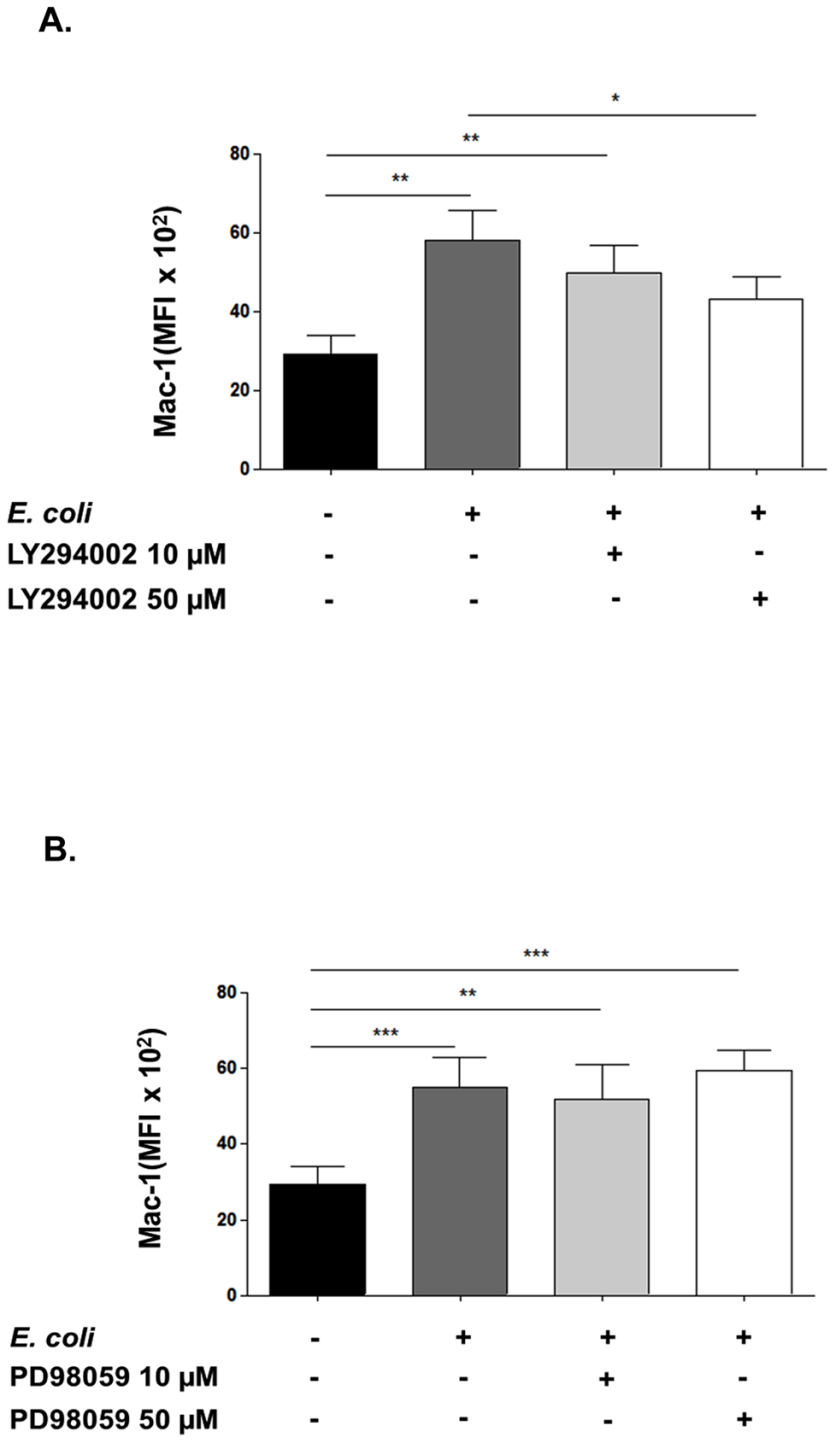
PI3K inhibition reduces Mac-1 activation. A. The PI3K inhibitor LY294002 and (B) the MEK1 inhibitor PD98059 (10 and 50 μM) were added to neutrophils for 30 minutes before stimulating the cells with opsonised *E. coli*. After 90 minutes the activation of Mac-1 was assessed by flow cytometry. Unstimulated and control *E. coli* stimulated samples were incubated with the highest concentration of carrier (DMSO) used. Data are mean ± SEM (n = 6). ** indicates p<0.01, *** p<0.001.

As adiponectin inhibited both PI3K-PKB and ERK signalling and also Mac-1 activation, we attempted to determine whether these two pathways were involved in Mac-1 conformational change in neutrophils stimulated with bacteria. Our results suggest that PI3K, but not ERK 1/2, contributes to regulating the activation of Mac-1 in response to bacterial stimulation, as pre-treatment with LY294002 (Fig. 4A) but not with PD98059 (Fig. 4B) caused a significant decrease in the activation of Mac-1.

### Adiponectin inhibits the F-actin increase in response to E. coli stimulation

**Figure 5:**
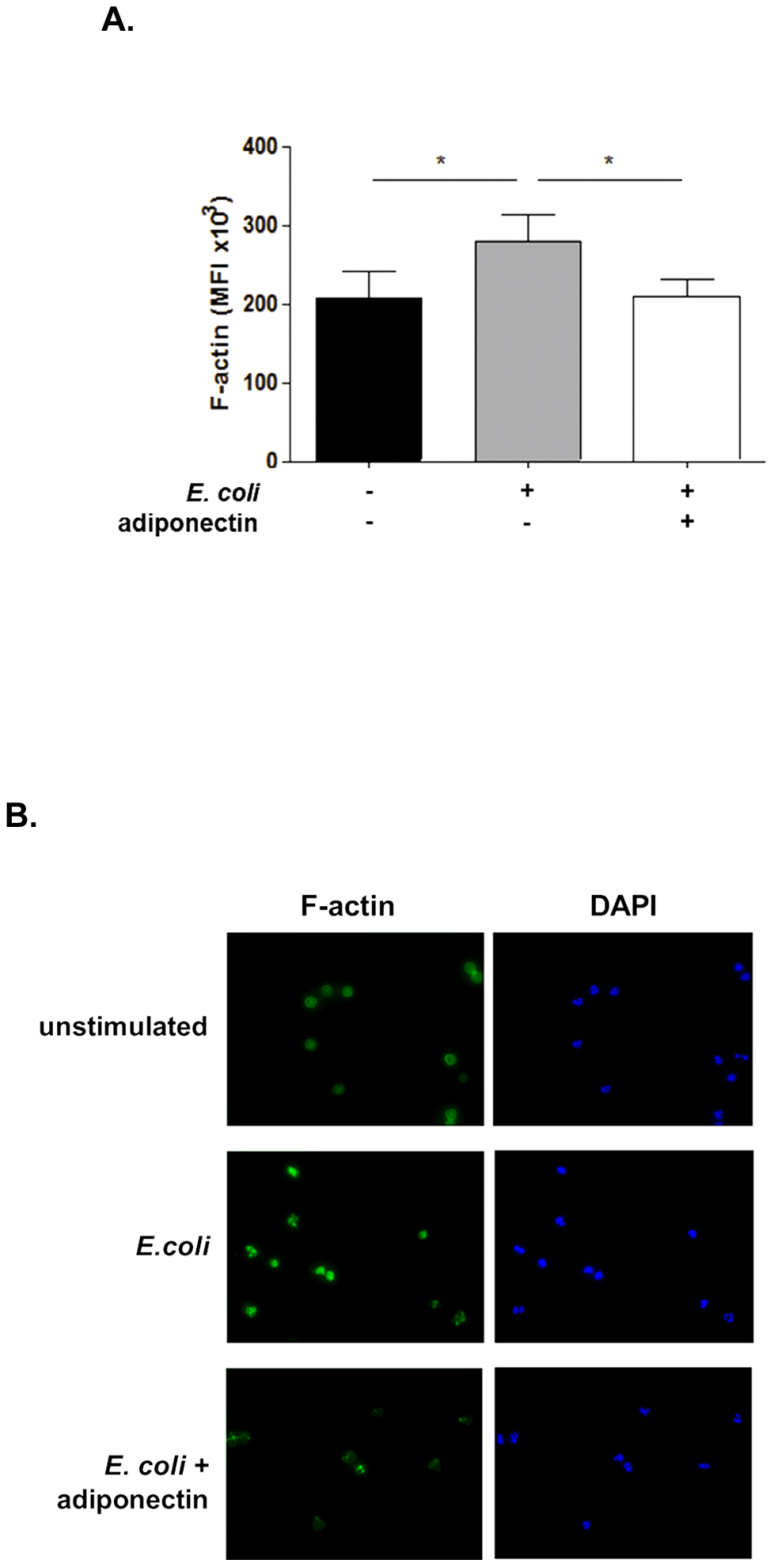
Adiponectin decreases actin polymerization in response to *E. coli* stimulation. A. Neutrophils were pre-incubated with adiponectin and stimulated with *E. coli* for 5 minutes, after which F-actin content was evaluated by staining with FITC phalloidin. Samples were analyzed by flow cytometry; data are mean ± SEM (n = 7). * indicates p<0.05. B. Representative images of F-actin in unstimulated and *E. coli* stimulated neutrophils with and without pre-treatment with adiponectin.

Actin polymerisation is required for bacterial uptake [35]. The extension of pseudopods is controlled by PI3K [26], [27] and ERK 1/2 has also been shown recently to play a role in regulating cytoskeletal modifications [36], [37]. As we found a decrease in PKB phosphorylation in the presence of adiponectin (Figure 3A), we proposed that the reduction in PI3K and ERK 1/2 activation could also have decreased F-actin generation in response to *E. coli*. F-actin staining with FITC phalloidin confirmed that adiponectin decreased actin polymerization after 5 minutes of stimulation with unlabelled bacteria (Figure 5), indicating a lack of pseudopod maturation and phagosome formation.

### Complement C1q does not prevent adiponectin effects on phagocytosis

**Figure 6:**
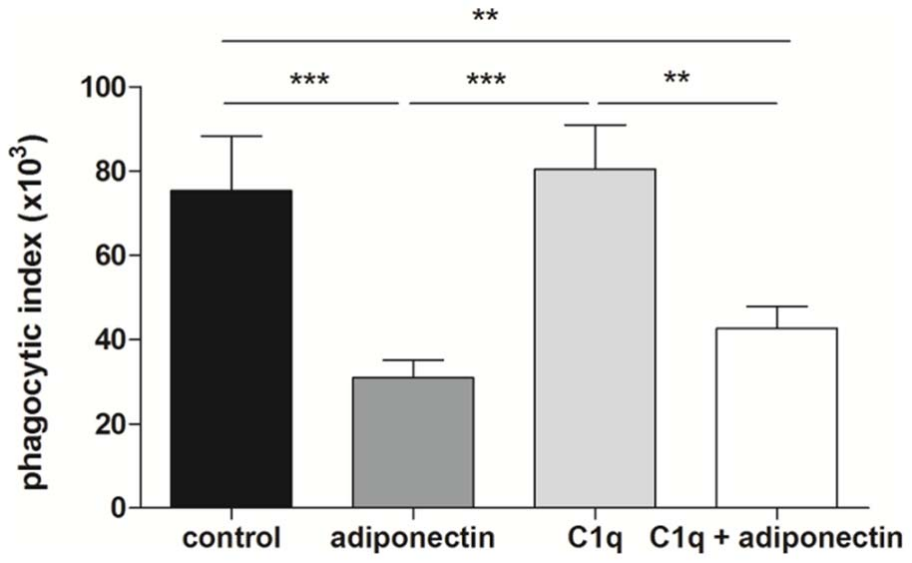
Adiponectin-mediated inhibition of neutrophil phagocytosis is not rescued by complement factor C1q. C1q (100 μg/ml) was added to neutrophils 15 minutes before the addition of adiponectin to saturate C1q receptors and phagocytosis of *E. coli* was then measured. Data are mean ± SEM (n = 6).

The adiponectin C-terminal domain shares homology with the serum complement protein C1q and the adiponectin-mediated inhibition of macrophage phagocytosis has been shown to be blocked by the use of a C1qRp antibody [18]. We therefore assessed whether inclusion of complement protein C1q protein would be able to block the adiponectin effect on phagocytosis. Neutrophils were pre-incubated with human serum C1q (at the physiological concentration of 100 μg/ml [38]) in order to allow binding to its receptors prior to the addition of adiponectin. However, the addition of C1q did not prevent adiponectin-mediated inhibition of neutrophil phagocytosis (Fig. 6).

## Discussion

The process of phagocytosis is central to the anti-microbial effects of neutrophils, representing the first step in the elimination of bacteria and resolution of infection. Full-length adiponectin has already been shown to impair neutrophil ability to kill pathogens by reducing the generation of ROS [12]. Here we have demonstrated that adiponectin also negatively affects neutrophil phagocytic ability in a concentration dependent manner. The inhibitory function of adiponectin was much greater on isolated neutrophils than in whole blood, which is most likely due to the presence of adiponectin in the blood, though we cannot exclude that the possibility that in whole blood adiponectin's effects are modified by other serum factors or immune cells.

We found that adiponectin did not change the expression of the phagocytic receptors CD11b, CD16 and TLR4 but it did significantly reduce the activation of the complement receptor Mac-1 in response to *E. coli* stimulation. Mac-1 binds to a range of ligands, the most relevant for phagocytosis being the opsonin iC3b. Accordingly, the binding of bacteria to the neutrophil surface was decreased. Mac-1 has also been shown to be involved in the engulfement of bacteria [39] and to enhance FcγR-mediated phagocytosis without affecting the binding [40], [41], hence Mac-1 reduced activation could also contribute to the decreased uptake of opsonised *E.coli*.

In addition, we revealed that adiponectin inhibited the phosphorylation of PKB and ERK 1/2 in response to bacterial stimulation. PKB phosphorylation represents a downstream measure of PI3K signalling, which sustains the phagocytic process [26], [27], [42]. ERK 1/2 also has a crucial role in neutrophil phagocytosis [37], [43], its activation being both downstream and independent of PI3K activation [27]. We were able to confirm their involvement in neutrophil phagocytosis by using the inhibitors LY29004 and PD98059. PD98059 treatment has been shown to lead to activation of AMPK indirectly by increasing the cellular AMP:ATP ratio [44]. However this should not contribute to decreased phagocytic ability as direct activation of this AMPK did not affect phagocytosis.

We also determined that the conformational change of Mac-1 was dependent on PI3K but not ERK 1/2 activation. Previous studies have shown that Mac-1 activation was mediated by cytoskeletal rearrangements [45] and the intracytoplasmic domain of CD18 is phosphorylated in stimulated neutrophils, contributing to the conformational rearrangement of Mac-1 [46]. The PI3K inhibitor LY29004 had a greater effect on neutrophil phagocytosis than it did on Mac-1 activation, suggesting that PI3K is involved in multiple aspects of the phagocytic process. For example, PI3K has also been shown to be indispensable for the correct closure of phagosomes [47].

ERKI 1/2 has been shown to be indispensable for phagocytosis of *Francisella tularensis*, acting downstream of the tyrosine kinase Syk, whose function is crucial for cytoskeletal rearrangements [37]. Importantly ERK 1/2, together with PI3K and other kinases, phosphorylates cortactin, a monomeric protein involved in actin polymerisation and lamellipodia formation [36]. Consistent with the reduced activation of PI3K and ERK 1/2, we observed a strong reduction in the content of F-actin in response to bacterial stimulation suggesting that adiponectin treatment inhibits the uptake of *E. coli* by blockage of both of these signal pathways.

AMPK is the main kinase previously reported to be activated by adiponectin [11], [12], however its activation does not mediate the decrease in phagocytosis induced by adiponectin, as AICAR treatment did not inhibit phagocytosis. This conclusion is supported by a previous report that showed AICAR actually increased neutrophil phagocytosis [48], though we did not see any enhancement of phagocytosis at the dose used here.

Adiponectin might also have inhibited neutrophil phagocytosis through its C-terminal C1q domain, which has been shown to act in an antagonistic manner in macrophages by Yokota and colleagues [18]. C1q is a serum complement factor whose main receptor is C1qRp, but it has been proposed to bind to other phagocytic receptors expressed by neutrophils, particularly CR1 [49]. To check whether adiponectin could decrease neutrophil phagocytosis by blocking bacterial binding to these receptors through its C1q-homologous domain, we pre-incubated the cells with a physiological concentration of C1q before the addition of adiponectin and before performing the phagocytosis assay, but we did not observe any improvement in neutrophil phagocytosis. Other groups have shown enhanced phagocytosis of apoptotic bodies mediated by C1q in human macrophages [17] and monocytic cell lines [17], [50], suggesting that C1q influences phagocytosis in a cell-specific manner.

**Figure 7:**
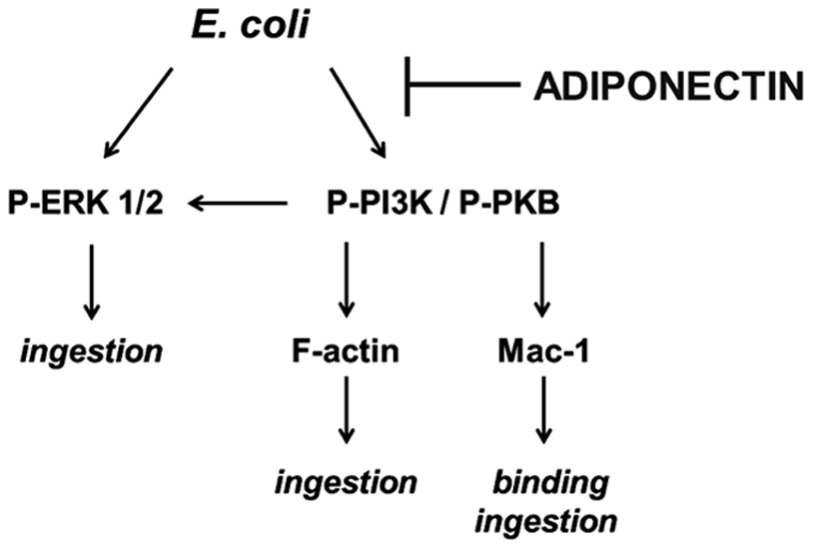
Proposed model for adiponectin inhibition of neutrophil phagocytosis.

In conclusion, these data demonstrate for the first time that adiponectin decreases neutrophil ability to phagocytose *E. coli*, a result observed both in whole blood and in isolated neutrophils. In Figure 7, we propose that adiponectin exerts its effect by decreasing the binding of bacteria to neutrophils through suppression of Mac-1 activation and both the bacterial binding and uptake are mediated by blockage of PI3K and ERK ½ signaling. These data add weight to the literature highlighting an immune modulatory role for adiponectin and importantly indicate that this could help to explain the increased incidence of infections seen in situations of raised circulating adiponectin, such as old age [51], [52]. The data are more difficult to interpret in the case of obesity, a complex condition which is characterized by higher risk of infection [53] but lower levels of adiponectin [8].

## Materials and Methods

### Ethics statement

The study was approved by the Birmingham East North and Solihull Research Ethics Committee (09/H1206/48) and all subjects gave their written informed consent before taking part in the study.

### Reagents and antibodies

Human recombinant full length adiponectin generated in HEK293 cells (HMW isoforms and hexamers, as specified in the datasheet) was purchased from Enzo Life Sciences (Farmingdale, NY, USA), with contamination from LPS certified to be less than 0.1 EU/µg purified protein. To ensure no artefactual effects from LPS contamination, Polymyxin B (10 μg/ml) (Millipore, MA) was added to the cells 30 min before treatment with adiponectin. Percoll, RPMI 1640 medium, L-glutamine, penicillin-streptomycin, bovine serum albumin (BSA), dimethyl sulfoxide (DMSO), human complement factor C1q, FITC phalloidin, protease inhibitor cocktail and all buffers and salt solutions were purchased from Sigma-Aldrich (Poole, UK). The MEK-1/ERK inhibitor PD98059 was from Cell Signaling Technology (Beverly, MA), the PI3K inhibitor LY29004 was from Millipore and the AMPK activator AICAR was purchased from Enzo Life Sciences. Unconjugated antibodies against AdipoR1 and AdipoR2 were from Phoenix Pharmaceuticals (Burlingame, CA), the unconjugated mouse anti-human CD11b (clone 2LPM19c), FITC conjugated CD16 (clone DJ130c) and isotype antibody FITC-conjugated mouse IgG1k were obtained from Dako (Ely, UK). FITC conjugated goat anti-rabbit secondary antibody was purchased from Southern Biotech (Birmingham, AL) and FITC conjugated goat anti-mouse secondary antibody was from Sigma-Aldrich. The anti-human CD284 (TLR4) (clone HTA125), the FITC conjugated mouse anti-human Mac-1 (activation epitope; clone CBRM1/5) and the APC-conjugated mouse IgG2a were obtained from eBiosciences (San Diego, CA). Antibodies against phosphorylated ERK1/2 (Thr202/Tyr204), total ERK 1/2, phosphorylated p38 MAPK (Thr180/Tyr182) and total PKB were purchased from Cell Signaling Technology, the antibody against total p38 MAPK was from Santa Cruz Biotechnology (Santa Cruz, CA) and the antibody against phosphorylated PKB (Ser473) was purchased from Millipore. Stabilized *E. coli*, FITC labelled and unlabelled, were obtained from Glycotope-Biotechnology GmbH (Heidelberg, Germany).

### Neutrophil isolation and treatment

Heparinised peripheral blood was obtained from healthy human donors and neutrophils were isolated by density centrifugation as previously described [54]. Briefly, 2% dextran solution was added to peripheral blood, the leukocyte layer was collected and neutrophils were purified by discontinuous Percoll density gradient centrifugation under endotoxin-free conditions. The purity of isolated neutrophils was determined by Giemsa staining (Diff-Quick, Baxter Healthcare, UK) and light microscopy and was routinely greater than 97%. For all studies neutrophils were resuspended in RPMI-1640 medium containing 2mM L-glutamine, 100 U/ml penicillin and 100 μg/ml streptomycin.

To evaluate whether adiponectin could influence neutrophil phagocytosis, either whole blood or isolated neutrophils were incubated with adiponectin or vehicle (sterile distilled water) for one hour prior to the assay. The protein kinase inhibitors/activators PD98059 (10 and 50 μM), LY294002 (10 and 50 μM) and AICAR (1 mM) were added to isolated neutrophils 30 minutes before the addition of the bacteria, whereas the complement factor C1q (100 μg/ml) was pre-incubated 15 minutes before addition of adiponectin.

### Measurement of neutrophil phagocytosis and binding of E. coli

Phagocytosis was assessed using the commercial kit Phagotest™ (Glycotope-Biotechnology GmbH, Germany) in whole blood according to the manufacturer's instructions: blood was incubated with *E. coli* for 10 minutes, erythrocytes were lysed and samples were analyzed by flow cytometry to distinguish uptake by neutrophils from that by monocytes. Using the same reagents provided by the kit but with further optimisation, phagocytosis by isolated neutrophils was also examined. Briefly, FITC labeled *E. coli* were opsonised with 10% autologous serum in Hanks balanced salt solution (HBSS) at 37°C for 30 minutes, after which they were washed in PBS and resuspended in medium at the same initial volume. All experiments with isolated neutrophils were performed in the absence of serum to exclude interference by adiponectin in the serum. 50 μl of neutrophils at 5×10^6^/ml were dispensed into a 96-well round bottomed plate (Sarstedt, UK) and *E. coli* were added (1∶40, 1∶20 or 1∶5 ratio between neutrophils and bacteria). The suspensions were incubated for 30, 60 or 90 minutes at 37°C, after which the assay was stopped by adding 100 µl of quenching solution to extinguish the FITC fluorescence of surface bound bacteria. A negative control was represented by the same suspension incubated at 0°C. The samples were transferred to FACS tubes and analyzed by flow cytometry (BD Accuri C6 Flow Cytometer, Accuri Cytometers Inc, Ann Arbor, MI) and the phagocytic index was determined. The phagocytic index was calculated as the percentage of the cells having ingested bacteria (FITC positive cells), multiplied by the mean fluorescence intensity (MFI) of the FITC positive population and divided by 100. Cytospins of the suspensions were also obtained and visualized using a LEICA DMI 6000 B microscope x63 objective (Leica Microsystems, UK).

To evaluate the binding of bacteria to neutrophil walls, suspensions were incubated at 4°C for 30, 60 and 90 minutes without final addition of quenching solution; samples were analyzed by flow cytometry, FITC negative cells were considered not to have bound bacteria.

### Measurement of surface receptor expression

Surface expression of adiponectin receptors (AdipoR1 and AdipoR2) and CD11b was measured by indirect immunofluorescence staining and negative controls consisted of staining with appropriate secondary antibodies alone. Adiponectin receptors were detected with unconjugated rabbit anti-human AdipoR1 and AdipoR2 (5 µg/ml) and CD11b expression was measured with mouse anti-CD11b (10 µg/ml). Nonspecific binding sites were blocked by addition of goat serum for 5 minutes before adding the secondary FITC conjugated goat anti-rabbit (2.5 μg/ml) or goat anti-mouse (5 μg/ml) antibodies. Surface expression of the activated form of Mac-1, CD16 and TLR4 were assessed by direct immunofluorescence staining (10 μg/ml for anti-Mac-1 and anti-TLR4, 4 µg/ml for anti-CD16); concentration-matched isotype antibodies mouse FITC IgG1 and mouse APC IgG2a were used as negative controls for Mac-1, CD16 and TLR4. Samples were analyzed by flow cytometry.

### Measurement of PI3K-PKB, ERK and p38 MAPK activation

Neutrophils were stimulated with unlabeled, opsonised *E. coli* for 10 minutes and spun at 4000 rpm for 4 minutes (MSE microcentrifuge) prior to resuspension in lysis buffer (20 mM MOPS, 50 mM NaF, 50 mM β-glycerophosphate , 50 mM Na_3_VO_4_, 1% Triton X-100, 1 mM DTT, 1 mM AEBSF and 1% protease inhibitor cocktail). Lysis of neutrophils was performed on ice for 30 minutes with occasional vortexing. The lysate was centrifuged at 13,000 rpm for 1 minute (MSE microcentrifuge) and the supernatant collected and combined with an equal volume of SDS-PAGE sample buffer (125 mM HCl pH 6.8, 5% glycerol, 2% SDS, 1% β-mercaptoethanol, 0.003% bromophenol blue). Lysates were boiled for 10 minutes, separated by SDS-PAGE and blotted onto PVDF membranes. Non-specific protein binding was blocked using 5% BSA. Membranes were incubated with primary antibodies to total PKB (1∶1000), phosphorylated PKB (1∶1000), total ERK 1/2 (1∶1000), phosphorylated ERK 1/2 (1∶1000), total p38 MAPK (1∶500), phosphorylated p38 MAPK (1∶1000), overnight at 4°C and with appropriate secondary antibodies (ECL^TM^ anti-rabbit or anti-mouse IgG; GE Healthcare, Sweden) for 1 hour at room temperature. Proteins were visualized by ECL according to manufacturer's instructions (GeneFlow, UK). Densitometric analyses were performed using Image J software.

### Measurement of actin polymerisation

Actin polymerisation was assessed by measuring F actin levels. Neutrophils were stimulated with unlabeled, opsonised *E. coli* for 5 minutes, after which they were subjected to fixation and permeabilisation using a Fix and Perm® kit (Life technologies, CA) according to the manufacturer's instructions. Neutrophils were incubated with FITC labeled phalloidin (1 µg/ml) for 30 minutes. After washing in PBS, samples were analysed by flow cytometry. Cytospins were also obtained and nuclei were stained with DAPI (Life Technologies) and cells were visualised using a LEICA DMI 6000 B microscope and x63 objective.

### Statistical analyses

Data were analysed using GraphPad Prism 4 software (GraphPad Software Ltd). Two-tailed paired Student's t-test was used to compare paired samples and repeated measures ANOVA was used to analyse more than two groups of matched samples, followed by Tukey's multiple comparison test. Results are expressed as mean ± standard error of the mean (SEM). A p value of less than 0.05 was accepted as significant.
